# Distinct Mechanisms Are Responsible for Nrf2-Keap1 Pathway Activation at Different Stages of Rat Hepatocarcinogenesis

**DOI:** 10.3390/cancers12082305

**Published:** 2020-08-16

**Authors:** Claudia Orrù, Andrea Perra, Marta Anna Kowalik, Sabrina Rizzolio, Elisabetta Puliga, Lavinia Cabras, Silvia Giordano, Amedeo Columbano

**Affiliations:** 1 Department of Biomedical Sciences, School of Medicine, University of Cagliari, 09042 Cagliari, Italy; claudia.orru@ircc.it (C.O.); ma.kowalik@unica.it (M.A.K.); lcabras@cnio.es (L.C.); 2 Department of Oncology, University of Torino, 10124 Torino, Italy; sabrina.rizzolio@ircc.it (S.R.); elisabetta.puliga@ircc.it (E.P.); 3 Candiolo Cancer Institute-FPO, IRCCS, 10060 Candiolo, Italy

**Keywords:** choline-devoid methionine-deficient diet, preneoplastic lesions, Nrf2 mutation, p62, Keap1

## Abstract

Activation of the Nrf2-Keap1 pathway, the main intracellular defense against environmental stress, has been observed in several human cancers, including hepatocellular carcinoma (HCC). Here, we assessed whether distinct mechanisms of activation may be involved at different stages of hepatocarcinogenesis. We adopted an experimental model consisting of treatment with diethylnitrosamine (DENA) followed by a choline-devoid methionine-deficient (CMD) diet for 4 months. The CMD diet was then replaced with a basal diet, and the animals were killed at 6, 10 or 13 months after DENA injection. Nrf2 activation occurred at early steps of hepatocarcinogenesis and persisted throughout the tumorigenic process. While *Nrf2* mutations were extremely frequent at early steps (90%), their incidence diminished with the progression to malignancy (25%). Conversely, while p62 was almost undetectable in early nodules, its accumulation occurred in HCCs, suggesting that Nrf2 pathway activation at late stages is mainly due to Keap1 sequestration by p62. We demonstrate that, in a model of hepatocarcinogenesis resembling human non-alcoholic fatty liver disease, *Nrf2* mutations are the earliest molecular changes responsible for the activation of the Nrf2-Keap1 pathway. The progressive loss of mutations associated with a concomitant p62 accumulation implies that distinct mechanisms are responsible for Nrf2-Keap1 pathway activation at different stages of hepatocarcinogenesis.

## 1. Introduction

Hepatocellular carcinoma (HCC) is the fourth most common cause of cancer-related death worldwide [1]. Unfortunately, our knowledge of the genetic/epigenetic alterations implicated in HCC initiation and progression is still fragmentary, and actionable driving genetic alterations have not been convincingly identified. In this scenario, HCC is probably one of the tumor types where a more complete understanding of the underlying genetic alterations could have a major impact on the development of new treatment strategies. *Nrf2*, also known as *Nfe2l2*, is of particular interest as its role in cancer development is conflictual [2,3,4]. While under normal conditions Nrf2 is negatively regulated and targeted for proteasomal degradation by Keap1 [5,6,7], following cytonuclear translocation, Nrf2 functions as a master transcriptional activator of genes encoding enzymes that protect cells from oxidative stress, xenobiotics and various drug efflux pump members of the multidrug resistance protein family [8].

In addition to this canonical pathway, Nrf2 activation also occurs through phosphorylation of the SQMST/p62 protein, which markedly increases its binding affinity to Keap1 [9] and results in the degradation of Keap1 via selective autophagy [10]. Phosphorylated p62 competitively abrogates the interaction between Nrf2 and Keap1, thus preventing Nrf2 degradation and allowing its nuclear translocation and activation, even in the absence of Keap1 alterations. As noted in liver injury and HCC, defective autophagy has been noted to result in the accumulation of p62-Keap1 aggregates and aberrant Nrf2 activation [11,12,13].

A number of studies have reported that point mutations in either the *Keap1* or *Nrf2* gene are often present in primary tumors [14,15,16,17]. As for human HCC, wide studies using whole-exome sequencing and TCGA-derived data have revealed mutations of either *Nrf2* (6 to 9%) [18,19] or *Keap1* (8%) [19,20], suggesting that the dysregulation of this pathway may play a relevant role in a subset of human HCCs. Notably, increased Nrf2 levels were associated with poor prognosis in HCC [21].

Increasing lines of evidence suggest that human HCC might arise as a consequence of increased oxidative stress associated with chronic non-alcoholic fatty liver (NAFL) and non-alcoholic steatohepatitis (NASH) [22,23]. Indeed, increased levels of reactive oxygen species (ROS) and lipid peroxidation products and decreased levels of antioxidant molecules have been observed in non-alcoholic fatty liver disease (NAFLD) patients [24]. In this context, the study of the Nrf2/Keap1 pathway is particularly relevant, given the high prevalence of NAFLD in the general population, and in particular in those affected by type 2 diabetes and obesity (approximately 30%) [25]. In mice models of diet-induced NAFLD, Nrf2 deficiency exacerbates NASH development while enhanced expression of Nrf2 attenuates fatty liver [26,27]. Nevertheless, activation of the Nrf2-Keap1 pathway has been shown to contribute to HCC development [18,21].

**Figure 1:**
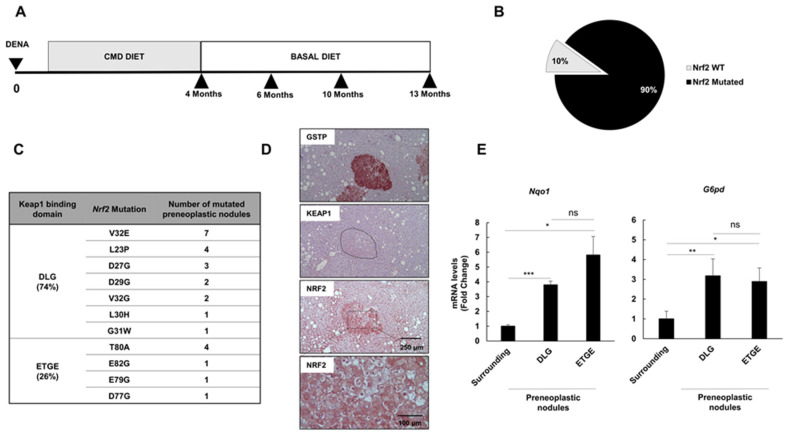
*Nrf2* mutations are an early event in choline-devoid methionine-deficient (CMD) diet-promoted preneoplastic hepatic nodules. (**A**) Experimental protocol; (**B**) Pie chart showing the percentage of glutathione transferase (GSTP)+ preneoplastic nodules displaying mutated or non-mutated (WT) *Nrf2* (27 mutated/30 nodules); (**C**) List of type and number of Nrf2 mutations identified in 27 nodules. All mutations are confined within the DLG (74%) or ETGE (26%) motif of exon 2 of the *Nrf2* gene. V32E represents the most frequent mutation at DLG (weak bond), while T80A is the most frequent at ETGE (strong bond); (**D**) Immunostaining of a GSTP+ lesion showing enhanced cytoplasmic and nuclear positivity to Nrf2. No significant increase in Keap1 could be detected in the same nodule (GSTP, NRF2 and KEAP1, 10×; inset NRF2, 20×); (**E**) *Nqo1* (left) and *G6pd* (right) mRNA levels in nodules carrying mutations in the DLG or ETGE motif. mRNA expression levels were assessed by qRT-PCR. Relative mRNA expression was calculated by using the 2^-∆∆CT^ method and *Gapdh* as endogenous control. Gene expression is reported as fold-change relative to surrounding tissue. Each bar represents mean ± standard error (SEM) of 4 to 17 samples per group. Student’s *t*-test was used for the evaluation of statistical significance. * *p* < 0.05; ** *p* < 0.01; *** *p* < 0.001; ns; not significant.

Our previous study showed that *Nrf2* mutations are extremely frequent in preneoplastic hepatic lesions generated in rat livers by a protocol consisting of a single injection of diethylnitrosamine (DENA) followed by feeding a choline-devoid methionine-deficient (CMD) diet [28]. This experimental model, like in NAFLD patients, is associated with steatosis, steatohepatitis and fibrosis [29]. Since only few preneoplastic lesions progress to HCC following CMD withdrawal [30], we investigated whether Nrf2 activation could represent a growth advantage for the progression of preneoplastic lesions to malignancy after CMD withdrawal (Figure 1A). In addition, we also explored whether mechanisms other than Nrf2 mutation could sustain the activation of the Nrf2/Keap1 pathway at different stages of the tumorigenic process.

## 2. Results

### 2.1. Nrf2 Mutations Are Very Frequent in Early Preneoplastic Nodules in Rats Fed a CMD Diet

We have previously shown that a single intraperitoneal dose of DENA followed by continuous feeding of a CMD diet for 4 months led to the development of preneoplastic nodules [28] positive for the placental form of glutathione transferase (GSTP), a well-known preneoplastic marker [31]. *Nrf2* somatic mutations occurred at a high frequency in GSTP+ preneoplastic lesions, demonstrating that mutation of this transcription factor is a very early event in hepatic tumorigenesis [28]. In the present study, we show that 4 months of CMD feeding following DENA administration resulted in a very high percentage (90%) of GSTP+ preneoplastic lesions carrying Nrf2 somatic mutations (27 out of 30) (Figure 1B). All of them were localized in exon 2 of the Nrf2 gene and confined within the two Keap1 binding domains: DLG (74%) and ETGE (26%) (Figure 1C). Since no interaction between Nrf2 and Keap1 might occur in the presence of these mutations [16], they are considered “bona fide” activating mutations. Activation of this pathway in preneoplastic lesions was indeed confirmed by enhanced Nrf2 NRF2 content and nuclear translocation in GSTP+ nodules (Figure 1D), which was associated with upregulation of *Nqo1 and G6pd*—two well established Nrf2 target genes [32,33] (Figure 1E). No significant difference in mRNA expression levels of *Nqo1* and *G6pd* was found between nodules carrying mutations in the DLG or in the ETGE domain. No difference in KEAP1 expression between preneoplastic lesions and surrounding liver was detected (Figure 1D).

### 2.2. Distinct Preneoplastic Populations Arise Following CMD Withdrawal

Since only a subset of preneoplastic lesions proceeds towards cancer following CMD withdrawal [30], we wondered whether Nrf2 activation could provide a growth advantage following withdrawal of the cytotoxic environment caused by feeding a CMD diet. To this aim, 28 rats were shifted to a basal diet and sacrificed 6, 10 or 13 months after DENA injection (Figure 1A).

**Figure 2:**
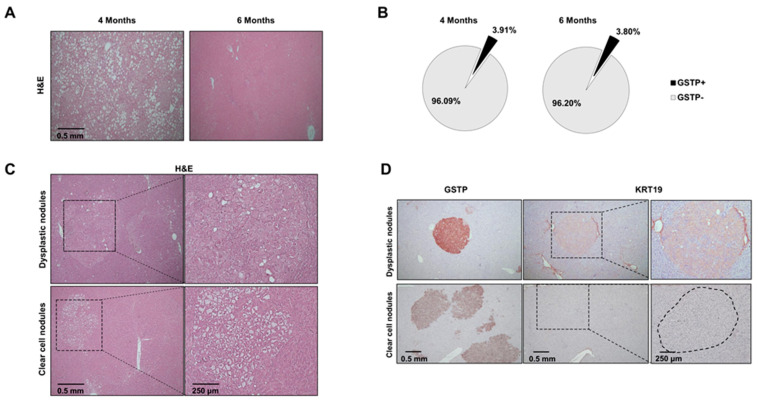
Emergence of dysplastic GSTP+ nodules displaying positivity for cytokeratin-19 (KRT-19) at 6 months after diethylnitrosamine (DENA) treatment. (**A**) Representative images of rat livers displaying huge accumulation of fat (left panel) or little steatosis (right panel) in rats killed at the times indicated above; (**B**) Pie charts showing the cytometric analysis, indicating the percentage of the GSTP+ and GSTP- area/total hepatic area in rats fed a CMD diet for four months (left pie) and in rats fed a CMD diet for 4 months and then shifted to a basal diet for 2 months (right pie); (**C**) Representative images of two types of nodules 6 months after DENA injection: dysplastic (top) and clear cell (bottom) nodules (H&E, 5×; inset 10×); (**D**) Immunostaining showing that a fraction of dysplastic nodules exhibited positivity for the stem/progenitor cell marker KRT-19. Magnification (10×) illustrates dysplastic (top) and clear cell (bottom) nodules positive and negative for KRT-19, respectively.

As expected, two months after CMD withdrawal (6 months after DENA treatment), livers no longer showed evidence of the massive fat accumulation observed at 4 months of continuous CMD feeding (Figure 2A). The hepatic area occupied by GSTP+ nodules was roughly similar to that observed before withdrawal (4 months) (Figure 2B). Notably, histological examination revealed the presence of two types of preneoplastic nodules (Figure 2C): clear cell and dysplastic nodules. Clear cell nodules displayed a clear (glycogen-rich) cytoplasm with a normal nuclear–cytoplasmic ratio, whereas dysplastic nodules were characterized by profound cellular alterations and a distorted nuclear–cytoplasmic ratio. Even though both types of nodules were GSTP+, a fraction of dysplastic, but not of clear cell, nodules displayed positivity for the putative stem/progenitor cell marker cytokeratin-19 (KRT-19) [34], suggesting a more aggressive phenotype [35,36] (Figure 2D). It is worth noting that no expression of KRT-19 was found in preneoplastic lesions of rats killed at 4 months, indicating that the acquisition of this marker in the CMD protocol occurs at later stages of the tumorigenic process, as also demonstrated in different rat and mouse hepatocarcinogenesis models [37,38].

Since no activation of the Nrf2-Keap1 pathway was observed in clear cell nodules, as demonstrated by the absence of NQO1 or G6PD staining in these lesions, we did not include them in the subsequent analyses.

### 2.3. Nrf2 Mutation Frequency in Preneoplastic Nodules Decreases Following CMD Withdrawal

**Figure 3:**
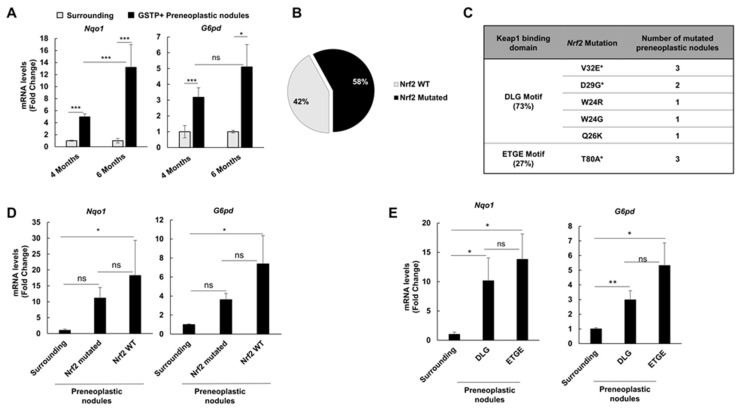
Nrf2 is also activated in the absence of an oxidative environment. (**A**) *Nqo1* (left) and *G6pd* (right) mRNA expression level in surrounding tissue and microdissected GSTP+ preneoplastic nodules. mRNA expression levels were assessed by qRT-PCR. Relative mRNA expression was calculated by using the 2^-∆∆CT^ method and *Gapdh* as endogenous control. Gene expression is reported as fold-change relative to respective surrounding tissue. Each bar represents mean ± standard error (SEM) of 5 to 21 samples per group. Student’s *t*-test was used for the evaluation of statistical significance. * *p* < 0.05; *** *p* < 0.001; (**B**) Pie chart representing the percentage (58%; 11/19 nodules) of *Nrf2* mutations in GSTP+ preneoplastic lesions; (**C**) List of Nrf2 mutations identified in GSTP+ nodules. All mutations are confined within the DLG (73%) or ETGE (27%) motif of exon 2 of the *Nrf2* gene. V32E is the only mutation at the weak bond (DLG) for Keap1, whereas T80A is the most frequent mutation in the strong domain for Keap1. Asterisks indicate mutations present at both 4 and 6 months; (**D**) *Nqo1* and *G6pd* mRNA levels in mutated and non-mutated (WT) preneoplastic nodules 6 months after DENA treatment; (**E**) Comparison of *Nqo1* and *G6pd* mRNA levels between DLG and ETGE motif mutations. mRNA expression levels were assessed by qRT-PCR. Relative mRNA expression was calculated by using the 2^-∆∆CT^ method and *Gapdh* as endogenous control. Gene expression is reported as fold-change relative to surrounding tissue. Each bar represents mean ± standard error (SEM) of 4 to 17 samples per group. ANOVA with Tukey post-hoc test was used for the evaluation of statistical significance. *p < 0.05; ** *p* < 0.01; ns, not significant.

To investigate the frequency of *Nrf2* mutations at later steps of hepatocarcinogenesis, we microdissected nineteen nodules and eight samples from the surrounding liver of rats treated with DENA + CMD and shifted to a basal diet for two months (6 months after DENA treatment). While most lesions were intensely NRF2-positive, KEAP1 staining was rarely observed (Figure S1). qRT-PCR analysis showed that the Nrf2-Keap1 pathway was strongly activated in GSTP+ preneoplastic lesions compared to the surrounding tissue, as revealed by the increased mRNA levels of *Nqo1* and *G6pd* (Figure 3A).

Unexpectedly, sequencing analysis showed that *Nrf2* mutations were present only in 58% of GSTP+ preneoplastic nodules vs. 90% of those identified at 4 months (Figure 3B). Interestingly, only 3 out of the original 11 mutations were maintained in preneoplastic nodules detected at 6 months after DENA administration (Figure 3C). Despite the decrease in the percentage of Nrf2-mutated GSTP+ nodules, the mutations observed at this stage maintained a ratio between the two Keap1 binding sites similar to earlier stages (73% DLG vs. 27% ETGE) (Figure 3C). Intriguingly, in spite of CMD withdrawal, activation of the Nrf2 pathway, evaluated by *Nqo1* and *G6pd* mRNA levels, resulted to be as strong as that observed at the 4-month experimental timepoint (Figure 3A). Unexpectedly, no significant difference in *Nqo1* and *G6pd* mRNA levels was detected between non-mutated vs. mutated nodules (Figure 3D). As observed above, at the 6-month experimental timepoint, there was also no difference in *Nqo1 and G6pd* expression between nodules carrying mutations either in the DLG or in the ETGE domain (Figure 3E).

### 2.4. The Frequency of Nrf2 Mutations Further Decreases in HCCs Occurring at 10 and 13 Months after DENA Injection

**Figure 4:**
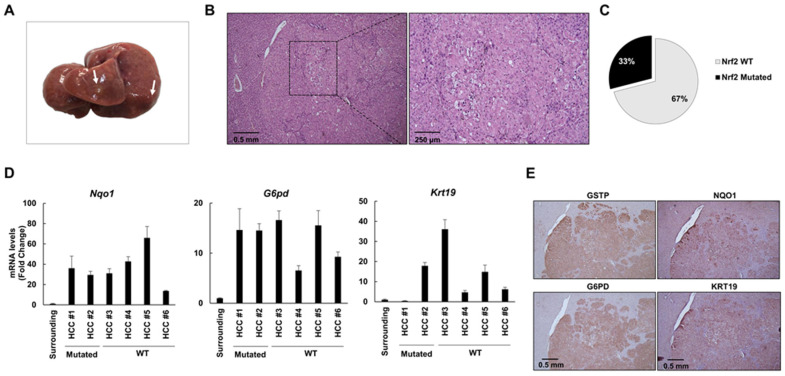
Nrf2 is activated in 10-month hepatocellular carcinomas (HCCs) in spite of low frequency of *Nrf2* mutations. (**A**) Macroscopic observation of livers from rats treated with DENA + CMD diet and shifted to a basal diet for 2 months (6 months after DENA treatment); arrows indicate the presence of HCC; (**B**) Histological observation of HCC (H&E, 5×; inset 10×); (**C**) Pie chart representing the percentage of HCCs exhibiting *Nrf2* mutations (33%); (**D**) *Nqo1* (left), *G6pd* (middle) and *Krt-19* (right) mRNA expression levels in surrounding tissue and microdissected HCCs. mRNA expression levels were assessed by qRT-PCR. Relative mRNA expression was calculated by using the 2^-∆∆CT^ method and *Gapdh* as endogenous control. Gene expression is reported as fold-change relative to surrounding tissue. Bar for surrounding tissue represents the mean ± standard error (SEM) of 4 samples; (**E**) Representative images of immunohistochemistry (IHC) of HCCs (5×) showing positivity for GSTP, NQO1, G6PD and KRT-19.

At 10 months after DENA injection, 4 out of 6 rats developed macroscopically evident tumors (Figure 4A). Histological analysis identified these tumors as HCCs displaying nuclear atypia with prominent nucleoli, increased nuclear/cytoplasmic ratio, apoptotic bodies and aberrant mitoses (Figure 4B, Figure S2). To investigate the extent of *Nrf2* mutations in HCCs developed 10 months after DENA treatment, we microdissected six HCCs and five surrounding tissues from five rats. Only 2/6 HCCs exhibited *Nrf2* mutations (33%) (Figure 4C). In both HCCs, we found the V32E mutation already identified at earlier stages (4 and 6 months). In spite of the decreased frequency of mutations, all tumors displayed a sustained activation of the Nrf2-Keap1 pathway, documented by NQO1 and G6PD overexpression (Figure 4D and E). Accordingly, the tumors developed at 10 and 13 months after DENA displayed a strong NRF2 cytoplasmic and nuclear staining (Figure S3A,B). HCCs bearing Nrf2 activation showed increased expression of the prognostic marker KRT-19, which in humans characterizes HCCs endowed with poor prognosis [35]. This observation was confirmed by KRT-19 immunostaining (Figure 4E). These results suggest that Nrf2 activation persists at late stages of hepatocarcinogenesis and strictly correlates to the expression of the prognostic marker KRT-19, and therefore, to the most aggressive phenotype.

**Figure 5:**
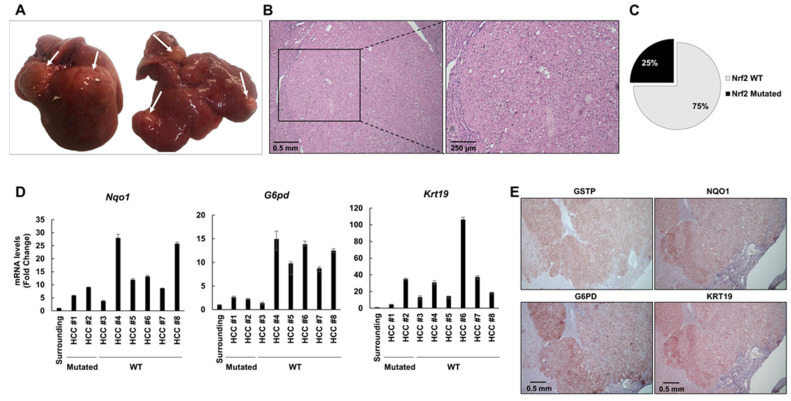
HCCs exhibit a low frequency of *Nrf2* mutations but sustained pathway activation at 13 months after DENA injection. (**A**) Macroscopic observation of livers from rats treated with DENA + CMD diet and shifted to a basal diet for 9 months (13 months after DENA treatment); arrows indicate the presence of HCCs; (**B**) Histological observation of HCCs (H&E, 5×; inset 10×); (**C**) Pie chart representing the percentage of HCCs exhibiting *Nrf2* mutations (25%); (**D**) *Nqo1* (left), *G6pd* (middle) and *Krt-19* (right) mRNA expression levels in surrounding tissue and microdissected HCCs. mRNA expression levels were assessed by qRT-PCR. Relative mRNA expression was calculated by using the 2^-∆∆CT^ method and *Gapdh* as endogenous control. Gene expression is reported as fold-change relative to surrounding tissue. Bar for surrounding tissue represents mean ± standard error (SEM) of 4 samples; (**E**) Representative images of IHC of HCCs (magnification 5×) showing positivity for GSTP, NQO1, G6PD and KRT-19.

Thirteen months after treatment with DENA, 6/6 rats developed multiple HCCs, mainly of the trabecular type (Figure 5A,B). Similar to what was observed at 10 months, *Nrf2* mutations occurred at a low frequency (3 out of 12 HCCs, 25%; Figure 5C): two of them (D29G and V32G) were confined within the DLG domain and the other one (T80A) within the ETGE domain. The occurrence of these mutations along the hepatocarcinogenic process suggests that they could confer a growth advantage to malignant cells.

Intriguingly, since activation of the Nrf2-Keap1 pathway, as detected by enhanced expression of *Nqo1, G6pd* and *Krt-19,* was found in all examined HCCs, it is independent of the presence of *Nrf2* gene mutation (Figure 5D). Positivity of HCC cells for these markers was confirmed by immunohistochemistry (IHC) (Figure 5E).

Since 8% of human HCCs are characterized by *KEAP1* mutations [20], we analyzed Keap1 exon 3, which is among the most frequently mutated. Results showed that *Keap1* mutations occurred at a very low frequency in HCCs (1 out of 14; W399R), suggesting that *Keap1* mutations are unlikely to be responsible for the constitutive activation of the Nrf2-Keap1 pathway in the majority of HCCs.

### 2.5. Keap1 Gene Silencing Is Not Responsible for NRF2 Activation in HCCs Devoid of Nrf2 Mutation

Overall, these results suggest that even though *Nrf2* mutations are very frequent at early steps of hepatocarcinogenesis, other mechanisms may be involved in the sustained activation of the Nrf2-Keap1 pathway observed during progression to malignancy.

**Figure 6:**
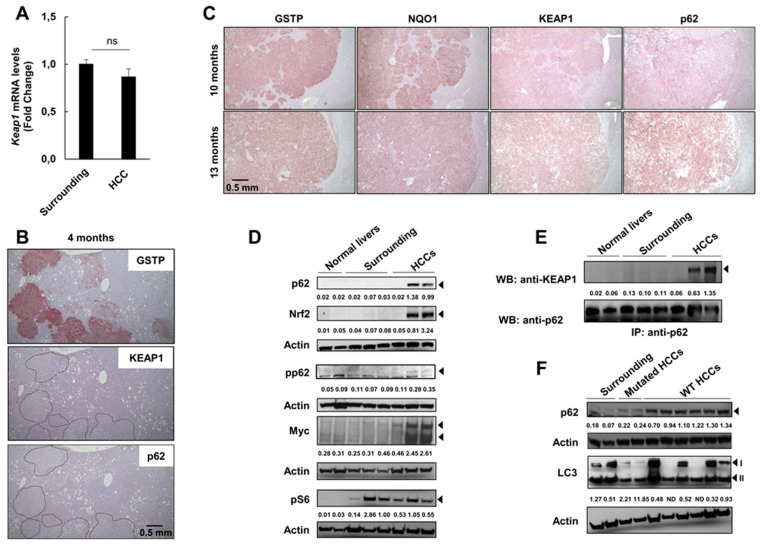
p62 accumulates in non-mutated HCCs. (**A**) *Keap1* mRNA expression levels in surrounding tissue and microdissected HCCs of rats sacrificed 13 months after DENA administration. Relative mRNA expression was calculated by using the 2^-∆∆CT^ method and *Gapdh* as endogenous control. Gene expression is reported as fold-change relative to surrounding tissue. Each bar represents mean ± standard error (SEM) of 6 to 9 samples per group. ns; not significant; (**B**) Microphotograph showing IHC of serial sections displaying no evidence of p62 or KEAP1 positivity in GSTP+ preneoplastic nodules at 4 months after DENA administration (5×); (**C**) Representative images of IHC of HCCs showing positivity for GSTP, NQO1, KEAP1 and p62 at 10 (top) and 13 (bottom) months after DENA injection (5×); (**D**) Western blot analysis showing p62, Nrf2, phospho-p62, myc and phospho-S6 levels in HCCs and in surrounding and normal liver. Numbers indicate the band intensity ratio between the protein of interest and the respective housekeeper protein (actin); (**E**) Co-immunoprecipitation assay. Total lysates from normal livers, surrounding tissues and HCCs were subjected to immunoprecipitation with anti-p62 antibody. The immunocomplexes were examined by immunoblotting with the indicated antibody; (**F**) Western blot analysis showing p62 levels and LC3 levels in WT and mutated HCCs developed 13 months after DENA treatment and in the surrounding liver tissue; accumulation of p62 is observed in non-mutated (WT) HCCs. Upper numbers indicate the band intensity ratio between the protein of interest and the respective housekeeper protein (actin); lower numbers indicate the band intensity ratio between LC3II and LC3I. See also Figures S7–S9 for detailed information about Figure 6D–F.

Several studies have shown that epigenetic regulation of the *Keap1* promoter results in the activation of the pathway [39]. Although *Keap1* promoter hypermethylation, negatively controlling gene expression, has been found in several cancers [40,41,42,43], not much is known about HCC. To investigate whether *Keap1* silencing could explain the activation of the Nrf2-Keap1 pathway observed in HCCs lacking *Nrf2* mutations, we performed qRT-PCR in HCCs at 13 months after DENA. As shown in Figure 6A, no difference in *Keap1* mRNA levels was found between tumors and the surrounding liver. Moreover, immunostaining displayed KEAP1 protein accumulation (Figure 6B), further demonstrating that *Keap1* gene silencing is not responsible for the sustained activation of the Nrf2-Keap1 pathway (evidenced by enhanced *Nqo1* expression) found in HCCs.

### 2.6. P62 Can Drive Activation of the Nrf2-Signaling Pathway at Late Stages of Tumorigenesis

Aberrant accumulation of p62 interferes with the interaction between Nrf2 and its inhibitor Keap1 [44]. The Keap1-interacting region (KIR) of p62 binds to Keap1 in a manner similar to the Nrf2 ETGE motif, thereby preventing Keap1 from trapping Nrf2, thus resulting in Nrf2 stabilization and activation [13,44]. As a consequence, the interaction between p62 and Keap1 contributes to progression to malignancy in an Nrf2-dependent manner [45,46,47]. To investigate the role of p62 in our system, we performed immunostaining on serial sections of preneoplastic nodules and HCCs positive for GSTP. The results showed that while no accumulation of KEAP1 and p62 was observed at early steps of hepatocarcinogenesis, whereby mutations of Nrf2 characterize the vast majority of preneoplastic lesions, a strong accumulation was found in most HCCs (Figure 6B,C and Figure S3A,B). P62 accumulation in HCCs, compared to surrounding and normal liver, was confirmed by western blot (WB) analysis (Figure 6D). Umemura et al. [48] have shown that in addition to activating Nrf2, p62 promotes hepatocarcinogenesis by increasing mTORC1 signaling and regulating c-Myc expression. Our results show that while p62 content positively correlated with an enhanced content of c-myc—a likely indicator of increased proliferation—a clear correlation between p62 and mTORC1 signaling, as determined by PS6 content, could not be determined (Figure 6D, Figure S4).

Since p62 phosphorylation in Ser 351 is important for Keap1/p62 interaction [9], we performed WB with specific phospho-p62 antibodies. As shown in Figure 6D, p62 was indeed phosphorylated in HCCs displaying its accumulation. To further establish the presence of p62/Keap1 interaction in HCCs, we performed co-immunoprecipitation experiments, which showed that Keap1/p62 interaction occurred in tumors but not in surrounding and normal liver (Figure 6E); as expected, increased p62 content was also associated with enhanced Nrf2 expression (Figure 6D).

Notably, p62 accumulation was evident in non-mutated HCCs but not in tumors carrying Nrf2 mutations or in the surrounding tissue (Figure 6F). Since p62 is degraded by autophagy [49], we investigated autophagic activity in these samples by measuring the conversion of LC3-I to LC3-II. Interestingly, a shift towards LC3-II was evident in mutated HCCs but not in most of the wild type tumors with p62 accumulation, suggesting an impairment of autophagy in the latter HCCs (Figure 6F, Figure S4).

Although the limited number of available samples and the variability among non-mutated (WT) tumors do not allow a definitive conclusion and further studies are required, the conversion of LC3-I to LCR-II is suggestive of an impairment of autophagy in WT HCCs (Figure 6F, Figure S4).

The finding of increased p62 in non-mutated HCCs suggests the possibility that the interaction between p62 and Keap1, with consequent inhibition of Nrf2 degradation, may be responsible for the activation of the Nrf2-Keap1 pathway at late stages of hepatocarcinogenesis.

## 3. Discussion

The most relevant findings stemming from the present study are the following: i) while *Nrf2* mutations are extremely frequent at early stages of hepatocarcinogenesis, their incidence decreases alongside malignant progression, in spite of sustained Nrf2-Keap1 pathway activation; ii) at late stages of the tumorigenic process, accumulation of p62, which competes with Nrf2 for Keap1 binding, contributes to Nrf2 activation.

In our work, we observed a constant decrease in *Nrf2* mutation frequency (from 90% to 25%) alongside the carcinogenic process. This finding suggests that while in the initial stage of hepatocarcinogenesis, *Nrf2* mutations confer a growth advantage by enhancing the survival capability of cells in a hostile environment (such as that generated by the CMD regimen), they might no longer be necessary, or could even be detrimental, for progression to malignancy when the liver is not exposed to the steatoinflammatory environment. Of note, only 3 mutations out of 11 persisted up to the HCC stage (V32E/G, D29G and T80A) (Figure S5), suggesting that they might be the most compatible with the development of liver cancer. Notably, D29 and T80 are among the five most frequent Nrf2 mutations identified in human tumors [50]. In contrast V32E/G, which is poorly represented in human cancers, is likely to be related to alkylating agent (such as DENA)-induced DNA transversions. Indeed, V32 mutations are the most frequent Nrf2 genetic alterations found in another DENA-induced hepatocarcinogenic model [51].

In our experimental model, Nrf2 mutations were more frequent (28%) than in human HCCs (6 to 9%) [18,19]; however, if we do not consider the DENA-associated V32 mutations, the frequency of the other mutations found in HCCs (D29G and T80A, which are due to a different mutation mechanism, namely transition) comes very close (11%) to that of human HCCs. Similarly, *Keap1* mutations occurred at approximately the same percentage as in humans (7% vs. 8%) [20].

Surprisingly, not much difference in Nrf2 transcriptional activity was observed between lesions presenting Nrf2 activating mutations or not. Interestingly, we observed p62 accumulation in the majority of HCCs devoid of Nrf2 mutations. The lack of p62 in preneoplastic nodules displaying Nrf2 mutation was somehow unexpected, as it is known that a loop exists between Nrf2 and p62 (p62 gene induction by oxidative stress is mediated by Nrf2 and, at the same time, p62 protein stimulates Nrf2 activation by disrupting Keap1-Nrf2 binding) [44]. Whether the mutated forms of Nrf2 are unable to interact with and induce p62 is unknown, and it will be an important issue to pursue.

P62 plays a critical role in normal and neoplastic cells as it is involved in several biological processes including autophagy, protein accumulation and apoptosis [44,45,46]. Among the processes modulated by p62 is the defense against oxidative stress that occurs through its binding to Keap1 and the subsequent release and nuclear translocation of Nrf2, leading to activation of the Keap1/Nrf2 system [45]. This enhancing antioxidative response of p62 is negatively regulated by tripartite motif-containing protein 21 (TRIM21), a ubiquitin E3 ligase, which directly interacts with and ubiquitylates p62 to regulate its sequestration and cellular redox homeostasis [52]. In contrast, the pro-oxidative effect of p62 is enhanced by the kinase mTORC1, which phosphorylates p62 to activate Nrf2 under stress conditions [9].

The finding that p62 accumulates at late stages of the hepatocarcinogenic process, when the frequency of Nrf2 mutations is strongly decreased, suggests that the interaction between p62 and Keap1, with consequent inhibition of Nrf2 degradation, is an alternative mechanism sustaining the activation of the Nrf2-Keap1 pathway. The reason why p62 accumulates in late stages of HCC development remains elusive. NRF2 is proposed to contribute to liver carcinogenesis by inhibiting death of initiated hepatocytes undergoing oxidative stress [53]. DENA-induced NRF2 activating mutations can thus confer an advantage in the early phases of hepatocarcinogenesis, as shown by the fact that NRF2 KO animals do not develop DENA-induced HCCs [28]. In DENA-treated mice, p62 accumulation is known to enhance the carcinogenic process since p62-deficient mice treated with DENA develop significantly fewer tumors than wild type animals [54]. It is possible that following DENA-induced initiation of hepatocarcinogenesis, once the size of the tumor increases and the availability of nutrients becomes limiting, p62 increase can represent an advantage for tumor cells, as it is able to drive autophagy and activate mTORC, NRF2 and MYC [48]. P62-driven hyperactivation of NRF2 in cells where this gene is already active due to mutations can lead to an excess of antioxidative activity, which thus counterselects NRF2-mutated hepatocytes.

Another open question is the reason for the difference in activating Nrf2 through genomic mutations compared to p62 accumulation. In this context, some hypotheses can be envisaged: i) Nrf2-activating mutations prevent the interaction between Nrf2 and Keap1, thus activating the Nrf2 pathway and leaving Keap1 available for interaction with other partners, such as WTX, PALB2, p62 and DPP3, which can contribute to Nrf2 activation [55]. Nrf2 positively regulates the gene expression of metabolic enzymes involved in the pentose phosphate pathway, purine nucleotide syntheses, glutathione syntheses and glutaminolysis. The activation of this transcription factor can thus redirect glucose and glutamine into anabolic pathways [33]; ii) the p62-Keap1–Nrf2 axis also promotes malignancy of HCC through similar, but not the same, metabolic reprogramming, as it enhances UDP-glucuronate and glutathione production, which have been shown to stimulate HCC growth [56,57]; moreover, the concomitant Nrf2 activation and p62-mTORC-mediated c-myc activation [48] could be another mechanism conferring a selective advantage to HCC cells in comparison to cells where NRF2 activation is due to activating mutations.

The correlation between p62 and Nrf2 has been also demonstrated in human HCC [45,58], where the presence of p62 correlates to a major risk of development of this cancer. Furthermore, our data mining analysis using TCGA showed a significant correlation between p62 gene expression and both Keap1 and Nqo1 (Figure S6), adding translational value to our present findings.

It is unclear why the triggering of this pathway could lead to different biological outcomes as a consequence of the modality of activation. Indeed, Keap1 can interact with and control the degradation of several partners. Therefore, the decreased availability of Keap1 in the presence of increased levels of p62 can modify the balance of expression of several interactors, contributing to cancer progression.

Independently of the responsible mechanism, the finding of the constitutive activation of the Nrf2-Keap1 pathway suggests its critical role in the process of hepatocarcinogenesis. This is in line with several studies reporting that Nrf2 activation is associated with HCC progression in animal models and that, in humans, it correlates to poor prognosis [4,21,58].

## 4. Materials and Methods

### 4.1. Animals and Treatments

Five-week-old male F344 rats were injected intraperitoneally with DENA (Sigma, St. Louis, MO, USA) at a dose of 150 mg/kg body weight. After a one-week recovery period, all rats were fed a CMD diet (Altromin, Vandoies, Germany) according to the formula used by Lombardi’s group [29]. Four months later, seven rats were sacrificed. The remaining animals were shifted to a basal diet and sacrificed at 6, 10 and 13 months after DENA treatment (for the experimental protocol, see Figure 1A). Animal procedures were performed according to procedures approved by the Ethics Commission of the University of Cagliari. Animals experiments and procedures were approved by the local Ethical Committee and by the Italian Ministry of Health (the ethical code is N.1247/15-PR), complying with national ethical guidelines for animal experimentation, and were conducted in accordance with *Principles of laboratory animal care* (NIH publication no. 85–23, revised 1985).

### 4.2. Histology and Immunohistochemistry

Immediately after sacrifice, liver sections were fixed in 10% formalin and embedded in paraffin or snap-frozen in liquid nitrogen. Paraffin-embedded tissues were cut into 4 μm sections, dewaxed and hydrated. Endogenous peroxide was inactivated using hydrogen peroxide. Slides were microwaved either in citrate buffer pH 6.0 (ab93678, Abcam, Cambridge, UK) or EDTA buffer pH 8.0 (ab64239, Abcam), except for GSTP, G6PD and p62 staining, followed by overnight incubation with the primary antibodies: GSTP and p62 (#311 and #PM045, respectively, MBL International., Woburn, MA, USA); KRT-19 (NB100-687, Novus Biologicals, Centennial, CO, USA); NRF2 (sc-722, Santa Cruz, Dallas, TX, USA); NQO1 (ab28947, Abcam); G6PD (sc-67165, Santa Cruz), KEAP1 (sc33569, Santa Cruz) and Cleaved-Caspase-3 (#9664, Cell Signalling, Danvers, MA, USA).

After washing, sections were incubated with the appropriate polymer DAKO Envision secondary antibody at room temperature. Signal was detected using the VECTOR^®^ NovaRED™ Peroxidase (HRP) Substrate Kit (Vector Laboratories, Burlingame, CA, USA). Sections were counterstained with Harris hematoxylin solution (Bio-Optica, Milano, Italy), passed through the dehydration process and covered. KRT-19-positive nodules were defined as lesions exhibiting a KRT-19-positive area of at least 5% of the total area of the preneoplastic lesion (the criterion commonly used by pathologists).

### 4.3. Cytometric Analysis

The area of GSTP-positive preneoplastic lesions was measured with ImageJ (NIH) according to Schneider et al. [59]. For each rat, two different sections from two distinct lobes were measured. Only nodules over 4000 μm^2^ were scored as GSTP-positive nodules.

### 4.4. Laser-Capture Microdissection (LMD)

GSTP-positive nodules were identified by immunohistochemistry (IHC) on 6 µm-thick frozen liver sections. Nodule microdissection was performed on 16 µm serial sections with a Leica LMD6000 (Leica Microsystems), as previously described [60]. Equivalent areas of surrounding tissue were similarly microdissected.

### 4.5. RNA Extraction and qRT-PCR

RNA was extracted from preneoplastic nodules and surrounding tissues after laser microdissection. For each experimental group, 20-30 nodules obtained from five to seven animals were used. For nodules, total RNA was extracted with the PicoPure kit (KIT0204, Thermo Fisher Scientific, Waltham, MA, USA), incubated for 30 min at 42 °C and stored at −80 °C until needed. For HCCs (*n* = 18) and their corresponding peritumoral tissues, RNA was extracted with the MirVana Kit (AM1560, Ambion). RNA was quantified using NanoDrop ND1000 (Thermo Fisher Scientific), while RNA integrity was assessed using Agilent Bioanalyzer 2100. Gene expression analysis was assessed by qRT-PCR using specific Taqman probes (Keap1, Rn00589292_m1; *Krt-19*, Rn1496867_m1; *G6pd,* Rn01529640_g1; Nqo1, Rn00566528_m1). Each sample was run in triplicate, and for the gene expression analysis, glyceraldehyde 3-phosphatase dehydrogenase (*Gapdh*) was used as the reference gene.

### 4.6. Western Blot and Co-Immunoprecipitation

Samples were lysed with cold EB buffer (20 mM Tris-HCl, 5 mM EDTA pH 8.0, 150 mM NaCl, 1% Triton X-100, 10% glycerol). One milligram of lysate was precipitated with 2 μg of P62 antibody (MBL-P045, MBL International, City, State if USA/Canada, Country) and protein A Sepharose beads for 2 h at 4 °C. Immunocomplexes were then washed three times with cold EB buffer and separated by SDS-PAGE. Western blotting was performed according to standard methods. Primary antibodies were as follows: p62 (MBL-P045, MBL International), pP62 (MBL-PM074, MBL), pS6 (4858S Cell Signaling), MYC (ab32072 Abcam), LC3 (L7543 Sigma-Aldrich St. Louis, MO, USA), NRF2 (sc-13032, Santa Cruz Biotechnology), KEAP1 (sc-515432, Santa Cruz Biotechnology). Actin was used as the loading control (A3854, Sigma-Aldrich). Band densitometry analysis was done with ImageJ software (NIH).

### 4.7. Gene Sequencing

To identify *Nrf2* and *Keap1* mutations, we analyzed a fragment corresponding to exon 2 of the rat *Nrf2* gene and a fragment corresponding to a part of exon 3 of the *Keap1* gene. cDNA was amplified (annealing 60 °C) with High-Fidelity Taq polymerase (Platinum Taq DNA Polymerase High Fidelity; Invitrogen, Carlsbad, CA, USA) and sequenced by fluorescence-based Sanger’s direct sequencing in a 3730 DNA Analyzer (Applied Biosystem, Foster City, CA, USA). The mutation nomenclature described in this study is given according to the numbering of the amino acids in the human protein sequence (Ensembl).

### 4.8. Statistical Analysis

Data are expressed as mean ± standard deviation (SD) or mean ± standard error (SE). Analysis of significance was done by Student’s *t* test or ANOVA with Tukey post-hoc test using the GraphPad software (La Jolla, California, CA, USA). *P*-values were considered significant at *p* < 0.05.

## 5. Conclusions

The main findings of this study can be summarized as follows: 1) using a multistage model of nutritional hepatocarcinogenesis, we have provided evidence that a progressive decrease in the frequency of *Nrf2* mutations occurs along with HCC progression; 2) in spite of this decrease, sustained Nrf2-Keap1 pathway activation characterizes all stages of the tumorigenic process; 3) activation of the Nrf2-Keap1 pathway at late stages is the consequence of accumulation of p62, which competes with Nrf2 for Keap1 binding, thus leading to an Nrf2-induced antioxidative response of cancer cells.

In conclusion, our study emphasizes the Nrf2-Keap1 pathway as a potential therapeutic target in HCC, and in the absence of specific inhibitors of Nrf2, suggests that p62 targeting may represent a promising therapeutic alternative. However, such an approach depends on the context/stage of the tumorigenic process, as benefits from interference with p62-mediated activation of the Nrf2-Keap1 pathway are likely to be achieved only at late stages of tumorigenesis.

## Supplementary Materials

The following are available online at https://www.mdpi.com/2072-6694/12/8/2305/s1, Figure S1: Immunostaining of a GSTP+ nodule developed 6 months after DENA treatment showing enhanced cytoplasmic and nuclear positivity to Nrf2. No significant increase in Keap1 could be detected in the same nodule (GSTP, NRF2 and KEAP1, p62; X10), Figure S2: Numerous apoptotic bodies in HCC developed 10 months after DENA treatment. Aberrant mitoses can also be observed (arrow) (H&E, X20; Caspase-3, X20), Figure S3: Numerous apoptotic bodies in HCC developed 10 months after DENA treatment. Aberrant mitoses can also be observed (arrow) (H&E, X20; Caspase-3, X20), Figure S4: Graphical quantification of the WBs (Western Blot) shown in Figure 6D (A), 6E (B) and 6F (C), Figure S5: Nrf2 mutations identified in preneoplastic and neoplastic lesions at different times after DENA treatment. Scheme illustrating the position of mutations in the Nrf2 Neh2 domain. All mutations are located in the LxxDQxDLG and DxETGE motifs responsible for Keap1 binding, Figure S6: Analysis of the Liver Hepatocellular Carcinoma (TCGA, Firehose Legacy) dataset to evaluate the correlation between p62 (referred to as SQSTM1) and NRF2, Keap1 and NQO1, Figure S7: Detailed information about Figure 6D, Figure S8: Detailed information about Figure 6E, Figure S9: Detailed information about Figure 6F.

## Abbreviations

CMD: choline-devoid methionine-deficient diet; GAPDH; glyceraldehyde 3-phosphate dehydrogenase; GSTP, glutathione S-transferase placental form; G6PD, glucose-6-phosphate dehydrogenase; HCC, hepatocellular carcinoma; KRT-19, cytokeratin-19; i.p., intraperitoneal; NAFLD, non-alcoholic fatty liver disease; NASH, non-alcoholic steatohepatitis; NQO1, NAD(P)H quinone oxidoreductase 1; PPP, pentose phosphate pathway; p62, SQSTM1/Sequestosome-1; qRT-PCR, quantitative reverse transcriptase polymerase chain reaction.
