# Supplementary material for: Distinct Mechanisms Are Responsible for Nrf2-Keap1 Pathway Activation at Different Stages of Rat Hepatocarcinogenesis
Source: Cancers (Basel). 2020 Aug 16;12(8):2305. doi: 10.3390/cancers12082305 (PMC7463589; doi:10.3390/cancers12082305)
Supplement: Supplementary file 1 [file cancers-12-02305-s001.pdf]

cancers

MDPI

Supplementary Files

Distinct Mechanisms Are Responsible for the Nrf2-Keap1 Pathway Activation at Different Stages of Rat Hepatocarcinogenesis

Claudia Orrù, Andrea Perra, Marta Anna Kowalik, Sabrina Rizzolio, Elisabetta Puliga, Lavinia Cabras, Silvia Giordano and Amedeo Columbano

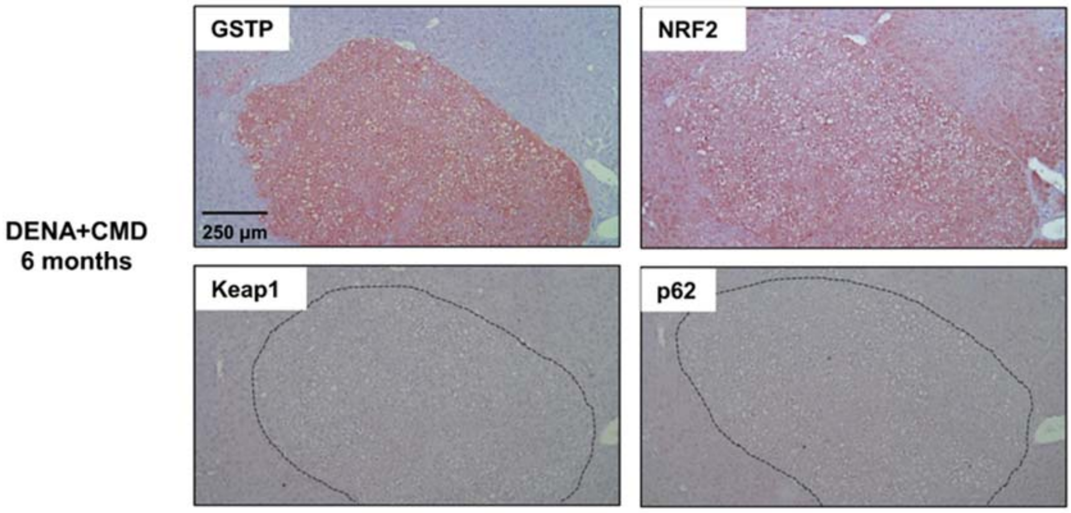

Figure S1. Immunostaining of a GSTP+ nodule developed 6 months after DENA treatment showing enhanced cytoplasmic and nuclear positivity to Nrf2. No significant increase of Keap1 or p62 could be detected in the same nodule (GSTP, NRF2 and KEAP1, p62; ×10).

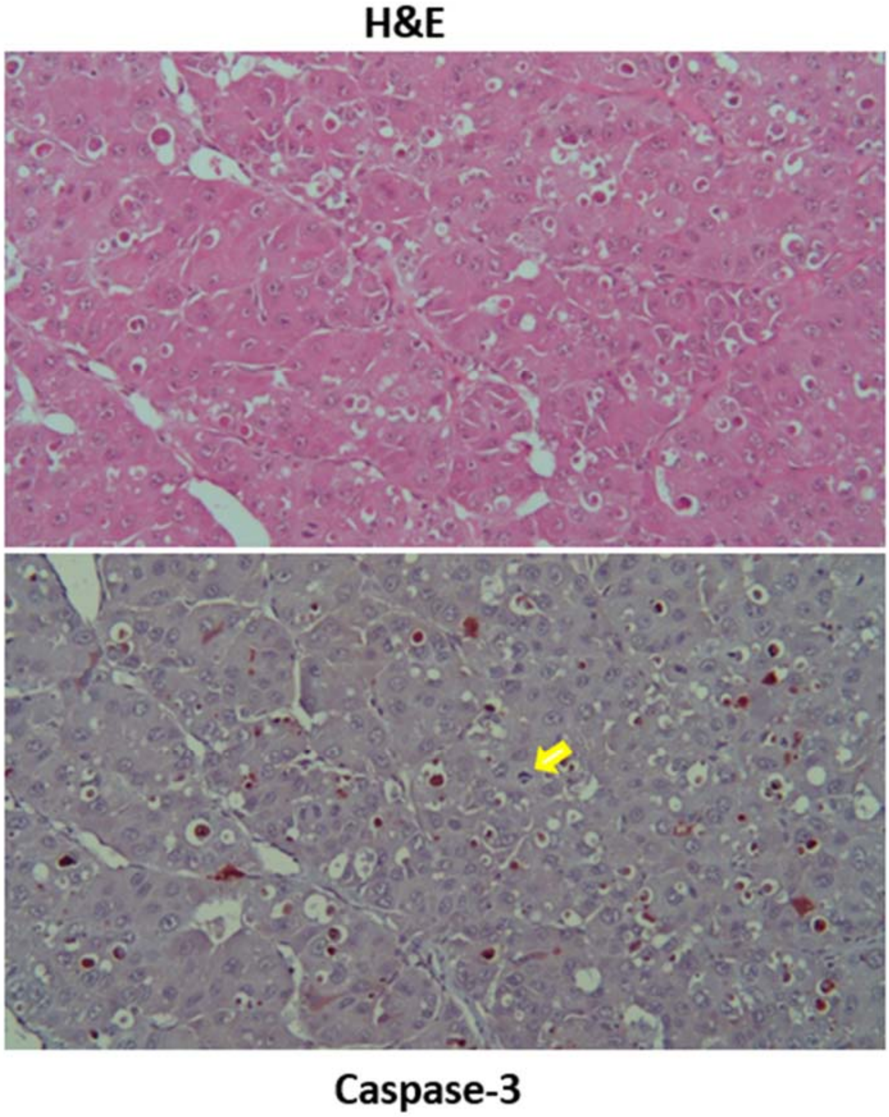

Figure S2. Numerous apoptotic bodies in HCC developed 10 months after DENA treatment. Aberrant mitoses can also be observed (arrow). H&E (×20); Caspase-3 (×20).

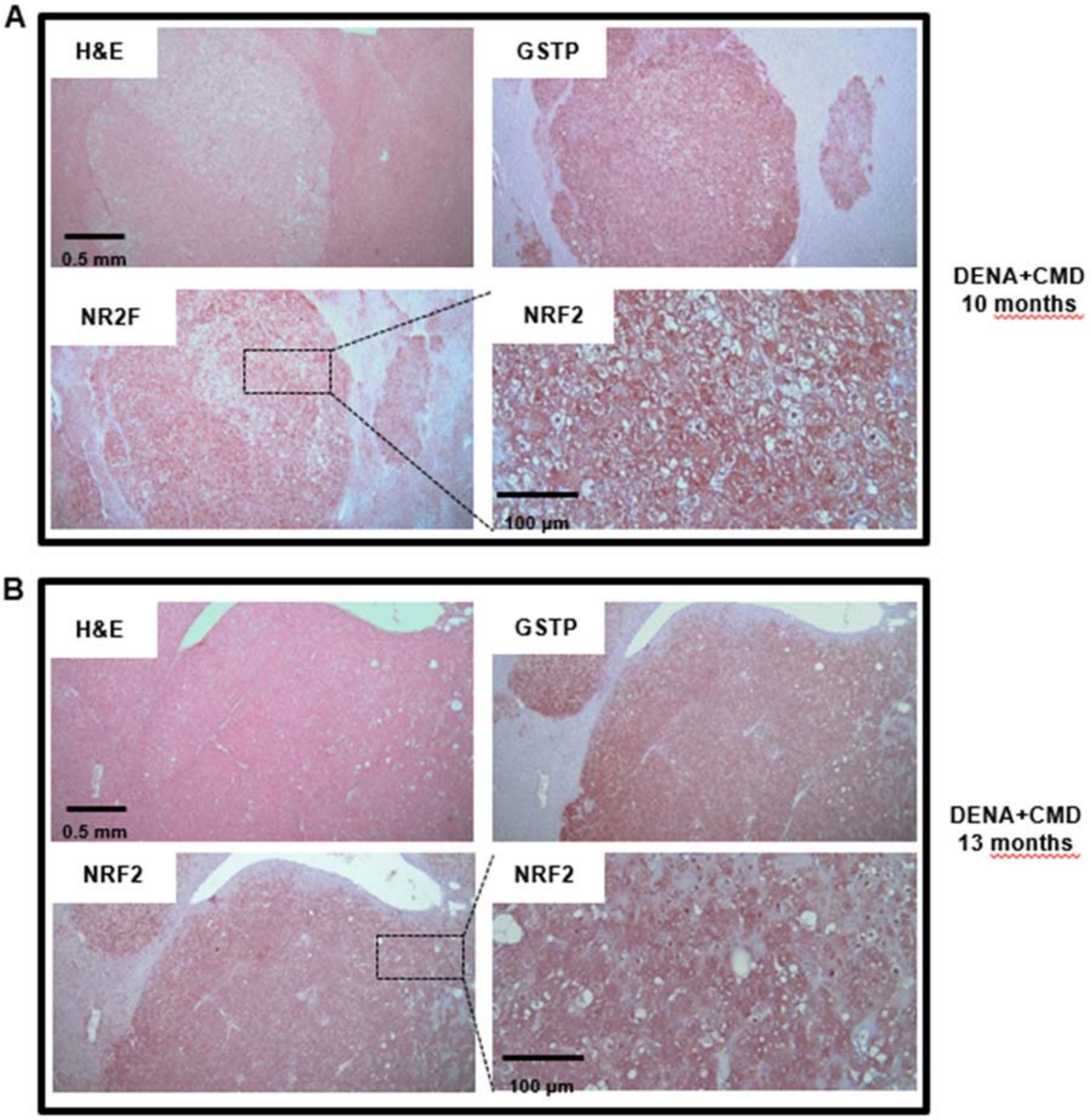

Figure S3. GSTP-positive HCCs developed (**A**) at 10 months or (**B**) 13 months after DENA showing enhanced cytoplasmic and nuclear positivity to Nrf2. (H&E, GSTP, NRF2 ×5; inset: NRF2 ×20).

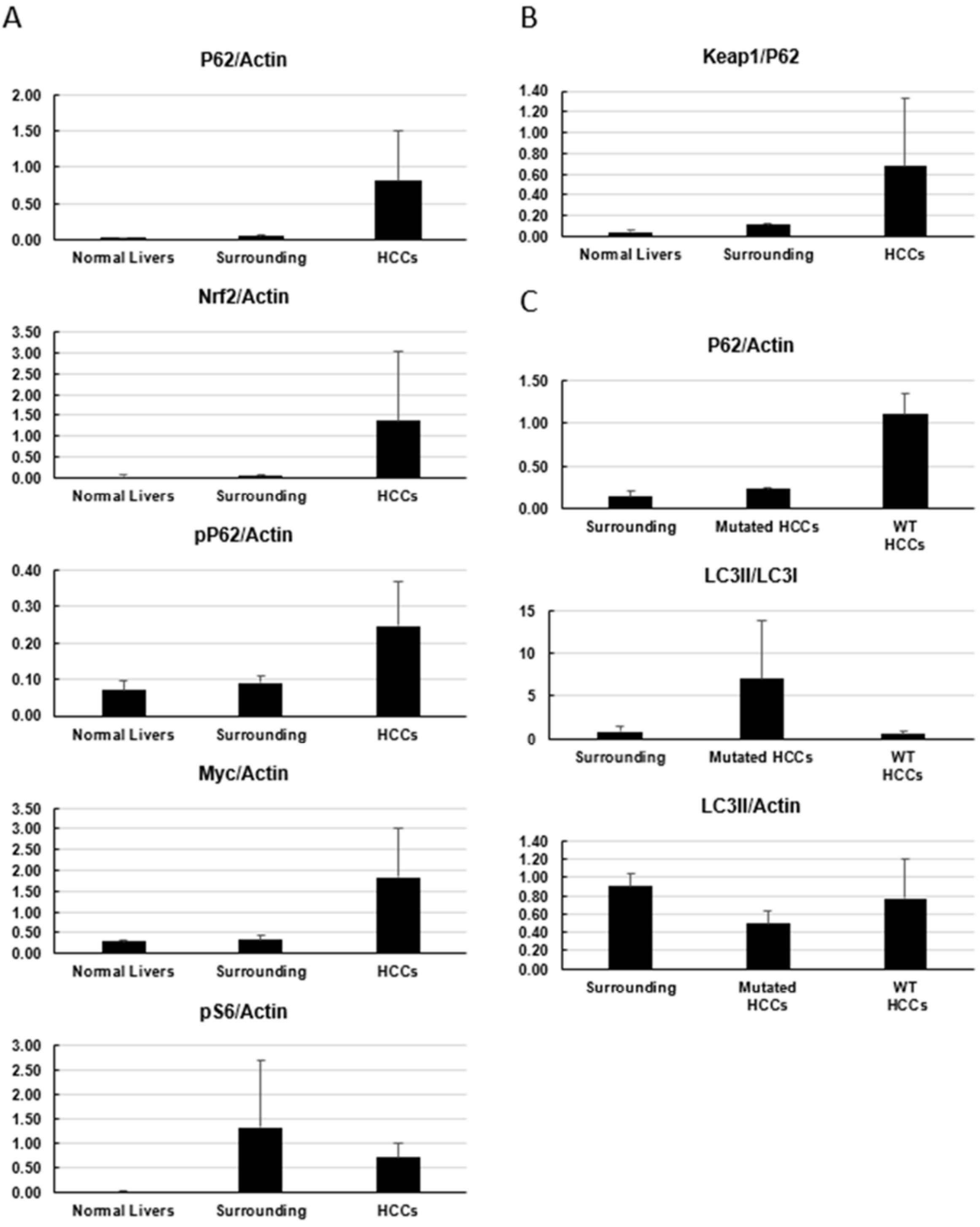

Figure S4. Graphical quantification of the WBs shown in Figure 6D (**A**), 6E (**B**) and 6F (**C**).

Nrf2 Mutation	4 Months	6 Months	10 Months	13 Months
V32E	x	x	x	
L23P	x			
D27G	x			
D29G	x	x		x
V32G	x			x
L30H	x			
G31W	x			
T80A	x	x		x
E82G	x			
E79G	x			
D77G	x			
W24R		x		
W24G		x		
Q26K		x		

Figure S5. Nrf2 mutations identified in preneoplastic and neoplastic lesions at different times after DENA treatment. Scheme illustrating the position of mutations in the Nrf2 Neh2 domain. All mutations are located in the LxxDQxDLG and DxETGE motifs responsible for Keap1 binding.

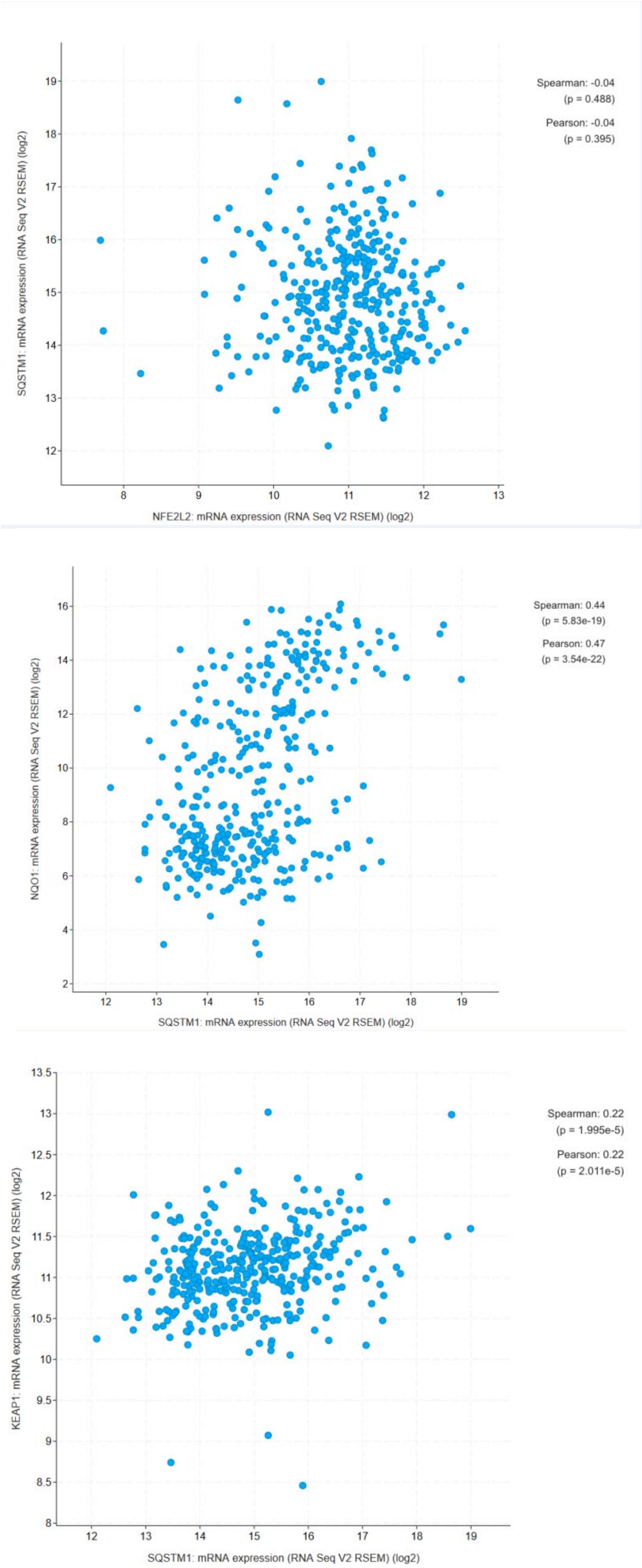

Figure S6. Analysis of the Liver Hepatocellular Carcinoma (TCGA, Firehose Legacy) dataset to evaluate the correlation between p62 (referred to as SQSTM1) and NRF2, Keap1 and NQO1.

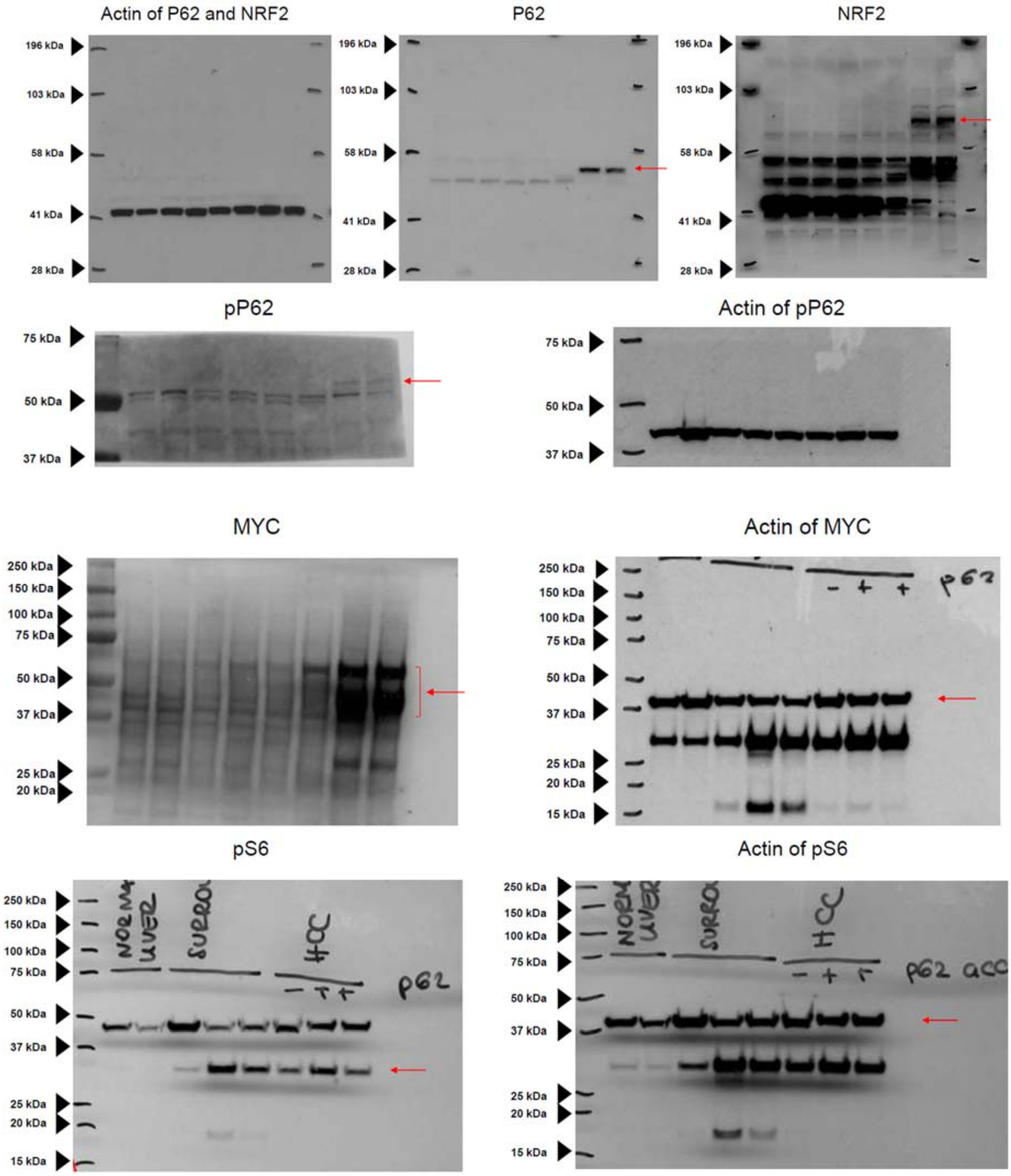

Figure S7. Detailed information about Figure 6D.

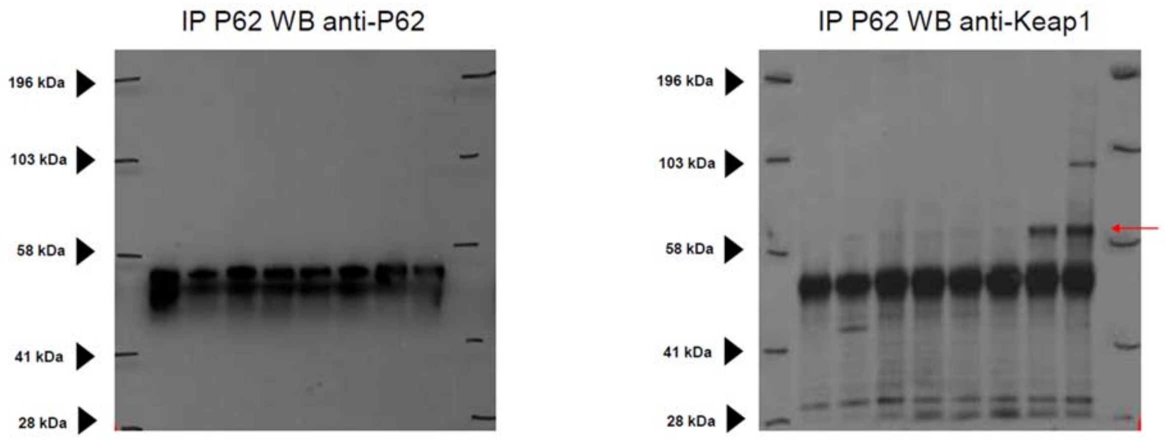

Figure S8. Detailed information about Figure 6E

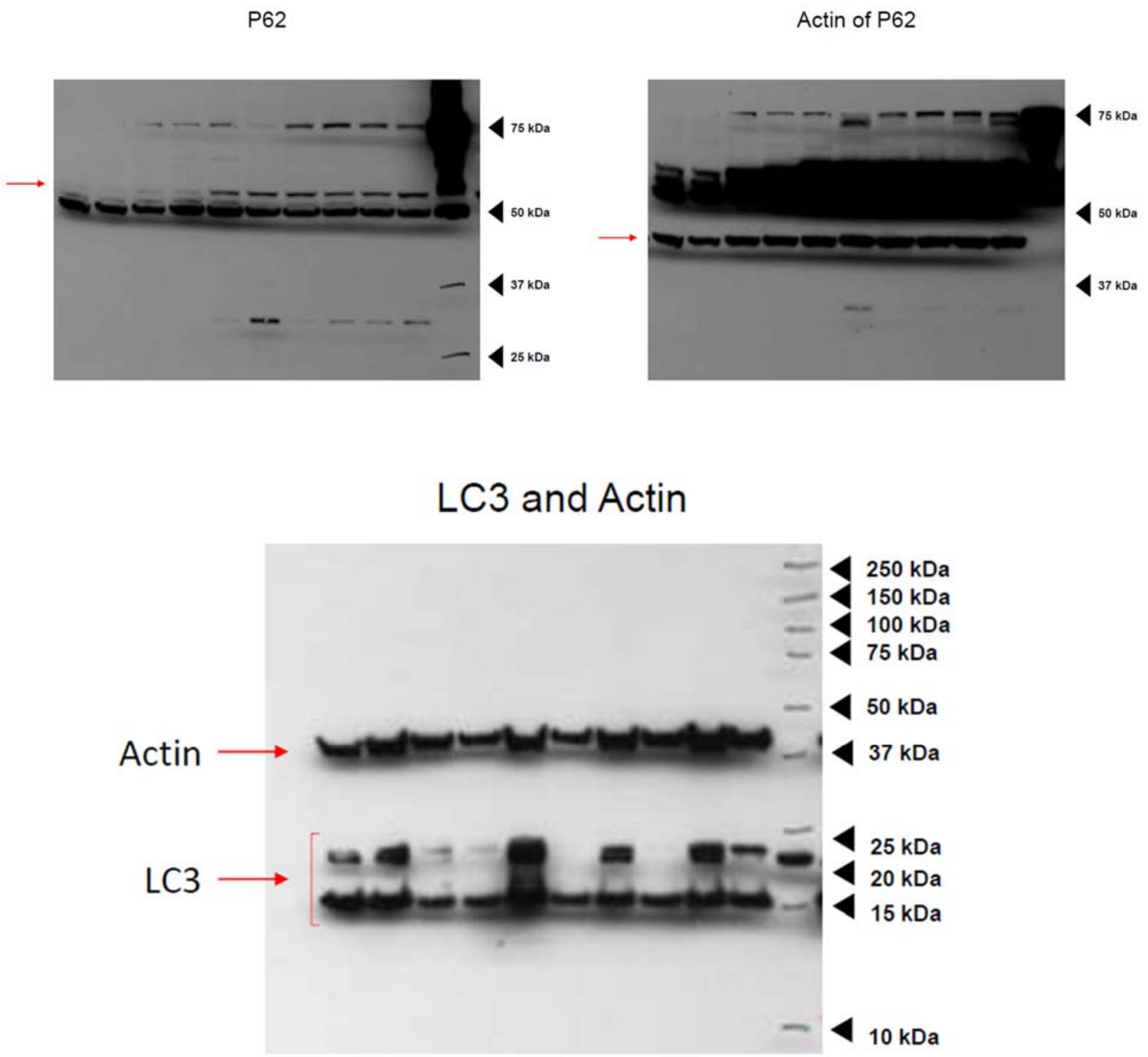

Figure S9. Detailed information about Figure 6F.

© 2020 by the authors. Licensee MDPI, Basel, Switzerland. This article is an open access article distributed under the terms and conditions of the Creative Commons Attribution (CC BY) license (http://creativecommons.org/licenses/by/4.0/).
